# A Role for Soluble IL-6 Receptor in Osteoarthritis

**DOI:** 10.3390/jfmk2030027

**Published:** 2017-08-02

**Authors:** Graham Akeson, Charles J. Malemud

**Affiliations:** 1 Department of Medicine, Division of Rheumatic Diseases, Case Western Reserve University School of Medicine and University Hospitals Cleveland Medical Center, Cleveland, OH 44106, USA; 2 Department of Medicine, University Hospitals Cleveland Medical Center, Foley Medical Building, 2061 Cornell Road, Room 207, Cleveland, OH 44106-5076, USA

**Keywords:** a disintegrin and metalloproteinase, cytokines, inflammation, interleukin-6, interleukin-6 receptor, osteoarthritis, signal transduction

## Abstract

Interleukin-6 (IL-6) is one of several pro-inflammatory cytokines present at elevated levels in the synovial fluid of individuals with confirmed clinical diagnosis of rheumatoid arthritis (RA) and osteoarthritis (OA). The mechanism of action of IL-6 was shown to involve its capacity to interact with a membrane-bound IL-6 receptor (mIL-6Rα), also known as the “classical” IL-6 pathway, or through its interaction with a soluble IL-6 receptor (sIL-6R) termed the “*trans*-signaling” pathway. Activation of downstream signaling is transduced via these IL-6 receptors and principally involves the Janus Kinase/Signal Transduction and Activators of Transcription (JAK/STAT) signaling pathway that is further regulated by glycoprotein-130 (gp130) interacting with the IL-6/mIL-6R complex. Phosphorylation of STAT proteins via JAK activation facilitates STAT proteins to act as transcription factors in inflammation. However, the biological function(s) of the sIL-6R in human chondrocytes requires further elucidation, although we previously showed that exogenous sIL-6R significantly suppressed the synthesis of neutrophil gelatinase-associated lipocalin (NGAL) in the immortalized line of human chondrocytes, C28/I2. NGAL was shown to regulate the activity of matrix metalloproteinase-9 (MMP-9), whose activity is crucial in OA for the destruction of articular cartilage. The “shedding” of sIL-6R from the plasma membrane is carried out by a family of enzymes known as A Distintegrin and Metalloproteinase (ADAM), which are also elevated in OA. In this paper, we have systematically reviewed the role played by IL-6 in OA. We have proposed that sIL-6R may be an important target for future drug development in OA by ameliorating cartilage extracellular protein degradation.

## 1. Introduction

At the pathophysiological level, human osteoarthritis (OA) can best be described as a systemic disturbance [1, 2] primarily involving large and small diarthrodial synovial joints [3]. Although the aetiopathogenesis of OA remains debatable, an examination of simple radiographs suggested that subtle changes in the anatomical structure of synovial joints, which can evolve during the ageing process, may be a contributing factor in the development of human generalized OA [4]. In addition, several components of cartilage and bone metabolism converge in OA which include genetic, mechanical, and microenvironmental stresses that are known to influence the progression of primary generalized and synovial joint-specific OA [5].

Just as important as genetic and mechanical stressors are to the development of OA was the recognition that a type of “non-classical” inflammation was also a significant contributing factor in the progression of OA [6–9]. Thus, accumulating evidence now appears to show that a chronic form of synovitis is a critical component that drives the progression of OA [3, 10–12]. Furthermore, the cellular infiltrates commonly associated with immune-mediated synovial tissue inflammation as seen in rheumatoid arthritis (RA), is evidenced in OA by the presence of activated T-lymphocytes [13–15]. There are also other cell-mediated inflammation biomarkers found in OA, including elevated levels of nitric oxide, prostaglandin E_2_, and neuropeptides [3]. Taken together with this and other evidence [3, 5, 7–9] these findings provide putative cellular and molecular mechanisms, as well as the pathophysiological underpinning for considering a role for immune cell and non-immune cell-mediated inflammation in OA together with the new data pointing to potential novel therapies for OA that could be developed based on those findings.

The inflammatory component of OA, as evidenced by chronic synovitis, is associated with a modulation of the chondrogenic phenotype. These changes include the upregulation of pro-inflammatory cytokine gene expression [16]; the upregulation of matrix metalloproteinase (MMP) gene expression [9, 17, 18] combined with a skewing of the ratio of the level of tissue inhibitor of metalloproteinases (TIMPs) to MMPs towards MMPs has also been considered as relevant; elevated expression of a disintegrin and metalloproteinases with thrombospondin motif (ADAMTS) genes [19]; and a disintegrin and metalloproteinase (ADAM) genes [20], the production of alarmins and Toll-like receptors [21], and an increased frequency of chondrocyte apoptosis [22]. These changes are likely to be arise from aberrations in signal transduction involving the mitogen-activated protein kinase (MAPK) and Janus Kinase/Signal Transduction and Activators of Transcription (JAK/STAT) pathways, negative regulators of JAK/STAT [23–26], and by those cytokines that activate the nuclear factor-κB (NF-κB) pathway [27–29].

Interleukin-6 (IL-6), in addition to other cytokines belonging to the IL-6 family of proteins, which include oncostatin M [30] and adiponectin (a member of the adipokine family) [9, 31], are among the most prominently elevated cytokines involved in the inflammatory response in OA. In that regard, it will be imperative that we further our understanding of the molecular mechanisms underlying the interaction between IL-6-type cytokines with the membrane form of the IL-6 receptor known as mIL-6Rα/gp130 and the soluble IL-6R form (sIL-6), as well as other respective membrane-bound receptors.

Of note, Livshits et al. [32] showed that an increase in the levels of circulating IL-6, as well as higher body mass index, had a predictive value in the development of confirmed radiographic knee OA. Furthermore, Blumenfeld et al. [33] studied thousands of female patients in the United Kingdom and found that single nucleotide polymorphisms (SNPs) in the IL-6 genomic region were associated with radiographic evidence of hand OA and an “osteoporosis-related phenotype” of the hand, suggesting that specific DNA motifs in the *IL-6* gene represented by these IL-6 SNPs contributed to the development of hand OA and osteoporotic-related changes in the hand.

With this in mind, we have briefly reviewed the current state of knowledge of how OA alters articular cartilage homeostasis and, in that regard, have explored the molecular mechanism by which the mIL-6R and/or sIL-6R can regulate articular chondrocyte gene expression in OA. Moreover, we propose a strategy by which sIL6R could be manipulated to ameliorate the progression of cartilage degradation in OA.

## 2. Cartilage Alterations in Osteoarthritis (OA)

The major changes occurring in OA of large diarthrodial synovial joints, such as hip, knee, and shoulder, involve significant alterations in articular cartilage and subchondral bone homeostasis, both of which are evidenced by radiographic or histologic evidence of osteophyte formation, chondrocyte senescence, and the increased frequency of apoptotic chondrocytes in articular cartilage as OA progresses [22, 34, 35]. However, the earliest measured changes in articular cartilage in OA also suggested a burst of hypermetabolic activity resulting from chondrocyte proliferation and an elevated production of proteoglycans and collagens [36]. At this stage of OA, subchondral bone cell homeostasis may also be perturbed [37], which is likely to be a precursor of, and eventually leads to, boney sclerosis. However, at its core, OA is characterized by an imbalance between anabolic and catabolic events leaning towards catabolism [38–40]. Thus, the significantly elevated levels of MMP, ADAMs, and ADAMTS activity in OA synovial fluid, which is caused by stimulation from pro-inflammatory cytokines, including TNF-α, IL-6, OSM, IL-17, and IL-1β, as well as an increased production of reactive oxygen species, lead to cartilage extracellular matrix protein (ECM) degradation and the loss of sulfated proteoglycans, collagens [10, 41], and accessory matrix proteins, such as fragmented fibronectin, from the tissue. In particular, OSM, in concert with TNF-α and IL-1β, have been implicated in the inflammatory process associated with OA wherein OSM mediated the degradation of aggrecan and hyaluronan, and where aggrecan degradation was associated with an increase in the low molecular weight G3 product of aggrecan [42]. Furthermore, Ni et al. [43] suggested that OSM may be involved in altering the metabolism of bone associated with OA progression. In addition, Greene and Loesser [44] showed that the chondrocyte in response to OSM and IL-1β, as well as growth factors such as IGF-1, may be responsible for initiating “cross-talk” between PI3K-Akt, MAP kinase, and the JAK-STAT pathways, which could provide the mechanism in OA for the differential responsiveness between anabolic and catabolic pathways in response to these factors.

This, in turn, not only results in a potent inflammatory response brought about by the egress of ECM protein fragments from cartilage into the synovial fluid, but also significantly compromises articular cartilage integrity, and also alters synovial joint biomechanical properties [45, 46].

## 3. “Classical” IL-6 Signaling Versus IL-6 *Trans*-Signaling

Interleukin-6 (IL-6) is one of several cytokine regulators of inflammation. At present, there are two principal mechanisms by which IL-6 is known to interact with its target cells. The “classical” pathway of IL-6 signaling involves membrane-bound IL-6 receptors (mIL-6R/mIL-6Rα) which associate with membrane-bound gp130 [47]. Gp130, when engaged by IL-6 bound to IL-6R, serves as a locus for a tyrosine kinase cascade, resulting in the activation of JAK/STAT and Src-family kinase signaling pathways [47–51], as well as ERK and PI3K/Akt/mTOR signaling [22, 44, 52]. However, only a limited number of cell types express membrane-bound mIL-6R, including hepatocytes, neutrophils, monocytes, macrophages, as well as naive and memory T-cells [53, 54]. IL-6R is also known to interact with ciliary neurotrophic factor [55].

IL-6R does not contain a signal-transduction domain. Gp130 serves as the signal transducer for IL-6R [47–51], and gp130 is a target for small-molecule inhibition of inflammatory pathways [56]. Circulating gp130 can bind to IL-6/sIL-6R complex, inactivating the complex, as well as sequestering the signaling molecules. This mechanism creates an IL-6 buffer. Thus, for classical or *trans*-signaling to occur, the concentration of IL-6 must be high enough so that the signal is not diluted by functional cytokine loss due to circulating gp130. This observation led to a therapeutic development with soluble gp130 employed as an IL-6 inhibitor [57].

Cells expressing membrane-bound IL-6R are the source of soluble IL-6R (sIL-6R) [58], and this soluble receptor is the mediator of the IL-6-*trans* signaling pathway. Approximately 80% of sIL-6R is produced by proteolytic cleavage of the membrane-bound IL-6R via ADAM 17 [59], and direct synthesis of the soluble receptor contributes to 20% of the circulating level of sIL-6R [59]. Once mIL-6R is released, IL-6 can bind to sIL-6R. This receptor-ligand pair interacts with membrane-bound gp130 which is expressed by a majority of cell types [60]. In that regard, once engaged, the gp130/sIL-6R/IL-6 complex induces protein kinase activity within the cell and activation of the JAK/STAT pathway [24], among other protein kinase pathways [61].

IL-6 does not require an IL-6 specific membrane-bound receptor to induce a response. This means that gp130 serves as the signal-transducing domain for both the classical- and *trans*-signaling pathways [62]. However, a novel mechanism of IL-6 signaling (termed “cluster signaling”) has been described for the development of T_h_17 cells in this pathway, membrane-bound IL-6/IL-6R/gp130 complex that is found on dendritic cells binds to and activates membrane-bound Gp130 on T-cells, promoting FoxP3 activation, which induces T_h_17 differentiation [63]. Thus, the proximal downstream response to IL-6 signaling does not seem to differ between cells based solely on the presence or absence of mIL-6R [64]. Accumulating evidence indicates that differences in phenotypic expression occur in response to “classical” IL-6 stimulation versus IL-6 *trans*-stimulation. Stimulation from the “classical” IL-6 pathway appears to primarily produce an anti-inflammatory effect [65], whereas *trans*-IL-6-stimulation predominantly results in a pro-inflammatory effect [66]. Of note, the homogenous nature of these signal transduction pathways can produce opposite phenotypes because the genomic targets of IL-6 signaling can vary based on cell type [67].

## 4. Metzincin Proteases Contribute to the Formation sIL-6R

IL-6 plays a pivotal role in many immune-cell-mediated responses in various disease states, including cancer [68] and arthritis [25]. As previously stated, the “classical” pathway of IL-6-mediated signal transduction involves membrane-bound IL-6R (mIL-6R; CD126). Thus, following the interaction of IL-6 with mIL-6R, two gp130 co-receptor molecules (CD130) are recruited to the IL-6/mIL-6R complex. In that manner, the recruitment of gp130 to mIL-6R provides the molecular mechanism for activating JAK/STAT signal transduction pathway [24]. Thus, STAT protein activation can provide the dominant signaling mechanism for increasing the level of pro-inflammatory cytokines in various arthritic disorders [18, 24, 25]. We have previously discussed the types of cytokines and growth factors that activate JAK/STAT signaling and the canonical IL-6 pathway, as well as the negative regulators of JAK/STAT signaling [24, 26] and the numerous downstream events which are coupled to STAT protein activation [24].

However, proteolytic cleavage of the mIL-6R protein is mediated by a class of enzymes termed metzincin proteases [69] which yields a soluble form of the IL-6R (sIL-6R). In addition, but to a lesser extent, the synthesis of mIL-6R can arise from an alternatively-spliced mIL-6R mRNA which can, therefore, result in the synthesis of a soluble form of IL-6R (sIL-6R) that can also interact with IL-6 [68, 70]. Importantly, Rose-John [70] defined sIL-6R as the critical component in IL-6-mediated signaling wherein the degree of IL-6-*trans*-signaling versus “classical” mIL-6R signaling regulated the apparent dichotomy between the pro-inflammatory and anti-inflammatory properties of IL-6.

**Figure 1:**
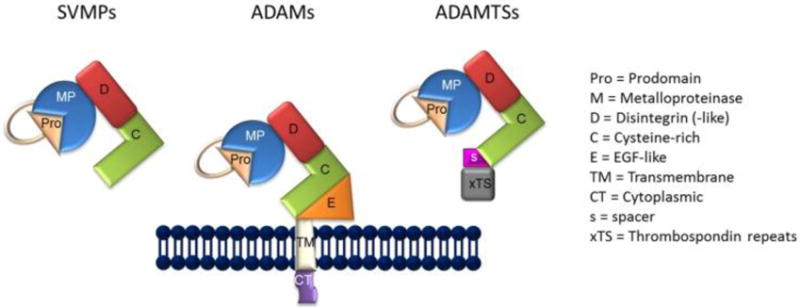
The general structure of snake venom metalloproteinases (SVMPs), A Distintegrin and Metalloproteinase (ADAMs), and ADAMTSs.

As previously indicated, the generation of sIL-6R is carried out by a molecular mechanism termed, “ectodomain shedding” or shedding [71–73]. Thus, shedding is facilitated by the ADAM class of metzincin proteases (Figure 1) [74]. As reviewed by Giebeler and Zigrino [74], the term “disintegrin” was originally employed to characterize a cysteine-rich RDG-domain in snake venom which was capable of binding to integrins, as well as by the capacity of snake venom to inhibit platelet aggregation in persons bitten by venomous snakes. In that respect, ADAMs are similar in structure to snake venom metalloproteinases (SVMP) (Figure 1) in their capacity to adhere to integrins, although the integrin binding sequence of the ADAMs differs from that of the SVMPs, the former containing mostly aspartic-acid sequences, with the exception of ADAM15 [73]. In the current context, deregulated shedding of membrane proteins by ADAM proteases has often been found in association with autoimmune disorders, cardiovascular diseases, neurodegeneration, cancer, infections, and a general state of inflammation [72].

In the case of mIL-6R, the enzymatic cleavage of mIL-6R occurs most notably by the action of either or both ADAM10 and ADAM17 [75, 76], although the assigned level of importance of ADAM10 in cancer and neurodegenerative diseases, such as Alzheimer’s disease [77], appears to make ADAM17 more crucial to its role in inflammation associated with autoimmunity [72]. In this regard, Schumacher et al. [78] showed that, after induction, the endogenous form of IL-6R from both human and mouse sources was shed due to the action of ADAM17, whereas constitutive shedding of IL-6R was, to a greater extent, mediated by ADAM10. In fact, it now can be stated with some certainty that ADAM10 mediates the constitutive release of sIL-6R from liver and hematopoietic cells which has been characterized as a slow process [78], whereas ADAM17 is more clearly involved with the regulation of sIL-6R release from neutrophils during both acute and chronic inflammation [75].

ADAM17 is a component of the plasma membrane [79, 80], and also called tumor necrosis factor-α converting enzyme (TACE). TACE, in addition to being capable of cleaving mIL-6R to produce sIL-6R, can also cleave ligands for ErbB, including transforming growth factor-α and amphiregulin. TACE is also implicated in the cleavage of some adhesion proteins, such as L-selectin and ICAM-1 [81, 82]. Also noteworthy was the finding that naturally-occurring isoforms of soluble gp130 were potentially endogenous inhibitors of sIL-6R-mediated signaling, in which case soluble gp130 would be an active component in the absence of “classical” IL-6 signaling [83]. In contrast to ADAM17, ADAM10 plays a significant role in the shedding of other substrates, including Notch, E-cadherin, epidermal growth factor, ErbB2, and inflammatory cytokines [84]. In addition, the shedding of Notch and CD23 by ADAM10 was also reported to be critical for lymphocyte development [85]. Thus, it was not unexpected that hydroxamate inhibitors of ADAM10 and ADAM17, exemplified by G1254023X and GW280264X, were evaluated as preferential inhibitors of cellular constitutive “ectodomain shedding” but without possessing the activity required to alter the capacity of ADAM10 and ADAM17 to induce “shedding” in response to phorbol esters, such as phorbol myristate acetate [86]. Importantly, in cell-based cleavage analyses, G1254023X blocked the constitutive release of mIL-6R, as well as the release of chemokines, CX3CL1/fractalkine and chemokine C-X-C ligand-16. These latter results were consistent with the reported role of ADAM10 in the release of soluble chemokine peptides [86].

A search of the PubMed database which was employed, in part, to select papers to be included in this review revealed several additional results of studies which investigated these hydroxamate compounds for their capacity to inhibit various ADAM-associated activities using cell-based assays [87–90]. Not unexpectedly, selective small molecular inhibitors of ADAM17/TACE have also been studied for their potential use as a future therapy for rheumatoid arthritis [91].

## 5. A Role for the ADAMs in OA?

Slightly more than a decade ago Campard et al. [92] showed that sIL-6R produced by shedding from peripheral blood-derived CD133^+^ cells purified to yield hematopoietic stem cells was abrogated by the ADAMs inhibitor, tumor necrosis factor-α protease inhibitor-1. This finding suggested that mIL-6R was not only the substrate for the shedding event but, moreover, that sIL-6R could also play a role in both the autocrine and paracrine loops which drives human stem cell development. In fact, in subsequent studies, ADAM17 was identified as the ADAM that mediated the shedding of mIL-6R [83], which produced sIL-6R that could be further processed by the activity of α-secretase into smaller peptides.

The shedding of mIL-6R in response to ADAM proteases also appears to be, in part, under genetic regulation since a single nucleotide polymorphism, rs2228145 in the *IL-6R* gene (i.e., the IL-6R Asp358Ala variant), conferred an increased susceptibility of mIL-6R to ADAM10- and ADAM17-mediated shedding [93]. Importantly, ADDAMS, ADAM10, and ADAM12 levels were found to be at increased levels in human OA along with other MMPs and the ADAMTS [20]. Furthermore, the finding of an elevated level of activity of ADAM17 in OA cartilage in which the rare double-secreted frizzled-related protein was expressed [94, 95] suggested that the origin of sIL-6R in OA cartilage was likely due to the “sheddase” activity of ADAM17. Importantly, Yan et al. [96] recently identified the natural protease required for the shedding of murine mIL-6Rα using hypomorphic ADAM10 or conditional ADAM17 knockout mice. In that paper, Yan et al. [96] showed that infection in these mice with *Listeria monocytogenes* caused the shedding of IL-6Rα by ADAM17 which was rapidly induced in leukocytes. However, CD4^+^-Cre-driven ADAM10 deletion in T-cells obtained from these mice did not alter mIL-6Rα shedding. This finding substantiated the role that ADAM17 played in producing sIL-6R following a challenge with an inflammatory stressor. Furthermore, a mechanism involving ADAM17 may also contribute to the increase in the biological availability and activity of sIL-6R in various types of arthritis [97] (also, see below).

We have previously shown that human chondrocytes enzymatically dissociated from OA knee cartilage synthesized neutrophil gelatinase-associated lipocalin (NGAL) in response to exogenous IL-1β [98]. Importantly, we have also previously shown that NGAL exists in a complex with MMP-9 (92 kDa gelatinase; gelatinase B) in synovial fluids sampled from OA patients undergoing joint replacement surgery [99]. Moreover, we showed that the MMP-9/NGAL complex was responsible for maintaining MMP-9 in its active state because when the MMP-9/NGAL complex was disrupted, MMP-9 activity was lost. We also found that exogenous sIL-6R, but not exogenous recombinant human IL-6 (rhIL-6), was a potent inhibitor of NGAL production in the immortalized human chondrocyte line, C28/I2 [100]. Furthermore, we reported that the combination of rhIL-6 and sIL-6R failed to suppress NGAL production, nor did exogenous tocilizumab (TCZ), a monoclonal antibody that blocks IL-6 signaling [101]. However, the combination of rhIL-6 and TCZ also inhibited NGAL production by C28/I2 human chondrocytes. Taken together with other data [102], we suggested that endogenous sIL-6R could potentially be manipulated so that it would behave as an inhibitor of NGAL production. This advance could limit the potential deleterious effects of MMP-9 on articular cartilage degradation in RA and OA.

## 6. Conclusions and Future Perspective

Meszaros and Malemud [5] reviewed the state of drug development for OA, which emphasized the fact that an OA-specific drug had yet to be developed. However, because the ADAMTS were also shown to be critical enzymes in OA cartilage for producing fragments of the sulfated proteoglycan, aggrecan, ADAMTS-4 and ADAMTS-5, in particular, have been targeted for potential future therapy of OA [103]. In that regard, IL-6 was shown to upregulate the expression of ADAMTS-4 [104]. Furthermore, the expression of the ADAMTS-4 gene was upregulated in vitro by the combination of IL-6 and sIL-6R-treated RA-fibroblast-like synoviocytes, whereas ADAMTS-5 was decreased. Of note, both ADAMTS-4 and ADAMTS-5 gene expression were dependent on IL-6/sIL-6R-mediated *trans*-activation of JAK/STAT. Therefore, in an important first step going forward it will be vital to critically and systematically evaluate the extent to which sIL-6R differentially regulates the activation of JAK/STAT signaling, as well as ADAMS/ADAMTS production, in human OA chondrocytes when those chondrocytes are compared to chondrocytes isolated from age-matched non-arthritis human cartilage. However, it will also be critical to consider that any pharmacologic approach to inhibiting ADAM proteases may produce important adverse side-effects. For example, because ADAM proteases also play a crucial role in developmental and regenerative processes [105], it will be incumbent to more accurately define both the temporal and spatial involvement of ADAM proteases in OA.
